# Monetary valuation of COVID-19 informal care: caregivers’ willingness to pay and willingness to accept

**DOI:** 10.1186/s12962-023-00437-9

**Published:** 2023-04-03

**Authors:** Vajiheh Ramezani-Doroh, Faride Karimi, Maryam Rangchian, Yadolah Hamidi

**Affiliations:** 1 Department of Health Management and Economics, School of Public Health, Hamadan University of Medical Sciences, Hamadan, Iran; 2 Modeling of Noncommunicable Diseases Research Center, Hamadan University of Medical Sciences, Hamadan, Iran; 3 Department of Clinical Pharmacy, School of Pharmacy, Hamadan University of Medical Sciences, Hamadan, Iran

**Keywords:** Informal care, Caregiver, Willingness to pay, Willingness to accept, Contingent valuation, COVID-19

## Abstract

**Background:**

Informal care can reduce hospitalization frequency and time, elevate bed turnover, and increase the health systems' capacity. This type of care has shown meaningful value in managing many cases through the COVID-19 pandemic. The present study aimed to identify determinants of monetary valuation of informal care and the burden of this care on the COVID-19 patients’ caregivers.

**Methods:**

Through a cross-sectional phone survey from June to September 2021 in Sanandaj city, the west of Iran, COVID-19 patients and their caregivers (Each Group No. 425) were separately interviewed. A simple probabilistic sampling method was applied. Two questionnaires were developed and used after validation. Monetary valuation of informal caregivers was done using Willingness to pay (WTP) and willingness to accept (WTA). Double hurdle regressions were used to determine related variables to WTP/WTA. R software was used for the data analysis.

**Results:**

The total mean (Standard Deviation) of WTP and WTA were $12.02(28.73), $10.30(15.43) USD. Most respondents put a zero value on informal care by WTA (243(57.18%) and WTP [263 (61.88%)]. Caregivers’ Employment, and being spouse/child of the care recipient increased the probability of reporting a positive value for WTP (p-value < 0.0001, p-value = 0.011 respectively) and WTA (p-value = 0.004, p-value < 0.0001 respectively). An increase in the number of caring days decreased the probability of reporting positive WTA (p-value = 0.001) and increased the mean of lnWTP (p-value = 0.044). Perceived difficulty in doing indoor activities and perceived difficulty in doing outdoor activities decreased lnWTA mean (p-value = 0.002) and lnWTP mean (p-value = 0.043) respectively.

**Conclusions:**

Increasing caregivers’ self-efficacy and facilitating their involvement in the caring process could be facilitated through flexible work status, educational programs, and interventions on decreasing their burnout.

## Background

In December 2019, an outbreak of pneumonia of unknown origin was reported in Wuhan, Hubei Province, China. The disease was identified as another type of coronavirus, and when the death toll exceeded 1000, the WHO registered it as a pandemic [1]. The spread of this virus has far-reaching consequences for many areas of life that cannot be ignored [2, 3]. For example, the health sector experienced a sharp increase in demand for healthcare due to its strong presence in the fight against the Coronavirus all around the world [3]. Many health systems were forced to prioritize patients for receiving hospital care, due to the lack of resources [4]. The high cost of using formal care is a major problem for patient’s families and the community [5, 6]. In this situation, many people stay at home and quarantine themselves to receive the required medical care by relying on family members, friends, or acquaintances. In addition, the lack of definitive treatment for this disease and the supportive role of existing therapies cause, after discharge from the hospital, many patients to undergo quarantine courses at home, and therefore they will need to receive informal care at home [7]. Family members and other acquaintances that take care of patients without getting paid are called informal caregivers. Related studies show that during the coronavirus disease of 2019 (COVID-19) pandemic, more than 25 percent of people have been forced to provide care to family, friends, or acquaintances through informal care [7, 8]. Home care can reduce the frequency and time of hospitalization in chronic patients, prevent unnecessary hospitalization, reduce the risk of nosocomial infections, increase patient comfort and safety, reduce treatment costs, elevate the turnover of the bed, speed up the discharge from the hospital and his/her return to normal life [8]. However, informal caregivers may face physical, social, and economic problems that require special attention. They also need special attention and psychological support in this regard [9, 10]. Informal caregivers require to be recognized and the issue of their health and well-being gets emphasized at the same time as the needs of patients [11]; utilizing some help in this regard might be helpful or, sometimes, necessary for them. Bastani et al.’s study, conducted in Iran in 2013, revealed that caregivers who received no help were more stressed [12]. Another research, performed in the same year, investigated the status of services provided by informal caregivers for the elderly living in homes in Iran. It showed that 53.9% of them had informal caregivers and 65.5% of the caregivers were women. 5.2% of the caregivers had a very heavy responsibility [13]. An article published in 2020 addressed the pattern of home care delivery, characteristics, mental health status, and challenges during the COVID-19 pandemic, among 765 adult Chinese. 25.1% of the participants had provided informal care, and of these, 18.4% had been forced to drop out of school or work to give this care. Most informal caregivers were young, women, and housewives. More than half of those reported mental health problems, and 37.2% said their daily lives had faced some challenges. Although most respondents reported being prepared to deal with the pandemic and being able to provide routine care, 49.5% of people said they were not able to deal with the potential dangers of COVID-19 [14].

Considering the importance of these cares and the challenges facing the providers of these cares, appropriate policymaking in this area is of significant importance, which in turn requires access to valid data. Then, the present study was conducted to investigate the determinants of informal care value for caregivers of patients with COVID-19 and compute the economic burden of informal care for these patients.

## Methods

### The aim, participants, study design, and setting

This study aimed to assess the economic burden of informal care, as well as to evaluate the effect of usual socio-demographic variables on it. A Contingent Valuation approach using WTP and WTA methods was applied to this end. The study was performed in Sanandaj city, from June to September 2021. The total number of COVID-19-infected individuals (with a positive result of the Polymerase Chain Reaction (PCR)) in every month in Sanandaj was extracted from the registry system and 425 patients were selected randomly. The sample size was calculated by the following formula:

$$n = {\left( {\frac{{Z1 - \frac{\alpha }{2}{\text{V}}}}{\Delta }} \right)^2}$$

where ∆ = difference between the real and estimated values of WTP/WTA = 0.1; Z = 1.96; V = CV = 1.

Assuming 10% attrition the final sample size was 425.

The participants included COVID-19 patients and their caregivers. Data gathering was conducted using phone surveying. In the first step, patients who were infected during the last month were selected randomly. Next, the interviewers called them, and if they consented to participate in the study the patient’s questionnaire was completed. Then the phone number of their main caregiver was taken. Next, the interviewers called the caregivers and if they were more than 18 years old and had consent to participate, the caregiver's questionnaire was filled out. For the patients who were under 18 years old, their parents answered the questions. If the caregivers refused to participate, the patient and his/her caregiver were excluded. Two questionnaires were developed: one for patients and another one for caregivers. The validity and reliability of the questionnaires were assessed. Content validity and face validity were assessed by experts. Reliability was calculated by Intraclass correlation coefficient (ICC); the obtained values were 0.8 and 0.82 for WTP and WTA respectively.

### Study instruments

#### Patients’ questionnaire

The patients' questionnaire had two parts. In the first section, the patient’s demographic and socio-economic characteristics were asked (age, sex, marital status, monthly family income, education, job, and having an aged person in the family). The second part was dedicated to the questions about their health status during the disease, history of receiving formal care in this period, number of hospitalization days, and their history of Intensive Care Unit (ICU) admission.

#### Caregivers’ questionnaire

The caregivers’ questionnaire had four parts. In the first section, the caregivers’ demographic and socio-economic characteristics were asked (age, sex, marital status, monthly family income, education, job, having an aged person in the family, and the relationship between patients and caregivers).

In the second part, questions about their health status during giving informal care (health status, getting COVID-19, and the patient’s dependency on the caregivers for daily activities) were examined.

The third part was dedicated to assessing the duration of caring for 4 activities’ categories (indoor activities, outdoor activities, personal care, and companying the patients to the formal health care centers), and the total care days. Because some activities may were done as routines before COVID-19 infection, the frequency and duration of each category were asked for two periods: before patients were infected with COVID-19 and during this disease.

The last part of the questionnaire addressed the most difficult activity for the caregivers and their WTP and WTA for this activity. To measure WTP and WTA an open-end format was used. Two scenarios were developed; in the first one, the caregivers declared their maximum WTP for a 1 h decrease in the most difficult activity that was provided by them, and in the second scenario they expressed the minimum amount that they were willing to accept for one additional hour of providing the most difficult activity. Since it was expected that some participants have no willingness to pay/accept money and some of them might be because of other reasons than economic explanations (protest zeros), the reasons for reporting zero WTP/WTA were also inquired. Zero responses that were arisen from ethical reasons were considered as the protest zeros.

### Data analysis

#### Descriptive and analytical analysis

Mean, standard deviation (SD), and frequency (percentage) were reported for descriptive statistics. Willingness to pay (WTP) and willingness to accept (WTA) perspectives were used to achieve the aim of this research. WTP addresses the maximum monetary amount that someone would be willing to pay for a good and WTA is the minimum amount of money someone is willing to take to give up a benefit or tolerate some harm [15].

Considering the high frequency of zero responses for WTP and WTA (and the high frequency of protest zeros) double hurdle regressions were applied. In the first hurdle, variables related to the probability of reporting a positive value for WTP and WTA were determined. In the second part of regressions, variables related to the values of the positive lnWTP (the natural logarithm of WTP) and lnWTA (the natural logarithm of WTA) were modeled. Since the distribution of WTP and WTA was highly skewed, their natural logarithm of them was included as the dependent variables in the second part of the regressions.

#### Calculation of the economic burden of informal care for the caregivers

The economic burden was computed in the following steps:

Predicting the positive amount for the protest zeros: since after removing obstacles caregivers who expressed a protest zero for WTP/WTA would have a positive amount for WTP/WTA, the positive amount for protest zeros was predicted. To this end, the expectations maximization method was used to predict the expected positive value of the protest zeros of WTP/WTA in 5 iterations.

Calculating the total time of providing care to the patients: first, for every activity, the duration of care was multiplied by its frequency to calculate the average daily time spent on each activity; this was performed for every period (before and during COVID-19). Next, the difference between these values (before and during COVID-19) was calculated. After that, the calculated difference was multiplied by the number of days of providing, to obtain the total time spent on every activity in all care days. Then, the values obtained for the 4 activity categories were summed for every individual to reach the time burden of informal care for every caregiver.

Monetary valuation of the total spent time: For the monetary valuation of the total spent time, the total time acquired in the previous step was multiplied by the reported WTP and WTA for every caregiver separately.

Economic burden of informal care: finally, the economic burden for the whole sample was obtained by summing the individuals' monetary valuation of the total spent time for every caregiver for both WTP and WTA, separately.

The statistical programming R software version 4.2 (http://www.R-project.org) was utilized to analyze the data. The significance level of 0.05 was considered.

## Results

The sex composition of patients was equal, however, most caregivers (289,68%) were female, with a mean (SD) age of 40.41(11.62) years. Most participants (256,60.24% of the patients and 260,61.18% of the caregivers) reported they were not employed during the time of getting/giving informal care. While most patients (280,65.88%) experienced a bad/very bad health status during COVID-19 infection, their caregivers assessed that their health status was moderate /good/very good (312, 73.41%) during giving care to the patients. Other characteristics of the participants are presented in Table 1.

**Table 1:** Patients’ and caregivers’ characteristics

Characteristics		Patients N (%)	Care givers N (%)
Gender	Male	212(49.88)	136(32)
	Female	213(5012)	289(68)
Age–mean (SD)		42.95(14.67)	40.41(11.62)
Marital status	Unmarried(single, widow, divorced)	124(29.18)	86(30.33)
	Married	301(70.82)	339(79.67)
Education level	Under bachelor	245(57.65)	260(61.18)
	Bachelor	137(32.23)	113(26.59)
	Master or higher	43(10.12)	52(12.23)
Family income	≤ $1302.30	323(76)	217(51.06)
	> $1302.30	102(24)	208(48.94)
Employment	No	256(60.24)	260(61.18)
	Yes	169(39.76)	165(38.82)
Health status	Bad and very bad	280(65.88)	113(26.59)
	Moderate/good/very good	145(34.12)	312(73.41)
Have a family member with the age of > 60	No	283(66.59)	305(71.76)
	Yes	142(33.41)	120(28.24)
Caregiver history in getting COVID-19	No	–	109(25.65)
	Yes	–	316(74.35)
Receiving formal care by patient	No	117(27.53)	–
	Yes	308(72.47)	–
Number of patients hospitalization days-– Mean (SD)		1.65(3.77)	–
Patients history in hospitalization in ICU	No	376(88.47)	–
	Yes	49(11.53)	–
Relationship of patient with caregiver	Spouse/child	**–**	298(75.12)
	other	**–**	127(29.88)
Living in the same place with the patient	No	**–**	50(11.76)
	Yes	**–**	375(88.24)
Dependency of patient to care giver	Completely/very much	**–**	306(72)
	Almost/a little/ at all	**–**	119(28)
The most difficult activity for care giver	Indoor activities	**–**	138(32.47)
	Outdoor activities	**–**	112(26.35)
	Personal activities	**–**	94(22.12)
	Tacking patients to the formal care centers	**–**	81(19.06)
Number of giving informal care days—Mean (SD)		**–**	15.50(7.77)
Average daily hours of giving informal care—Mean (SD)		**–**	5.29(2.98)

Up to the caregivers, the most difficult activity was indoor activities (32.47%). The mean (SD) of days that the caregivers provided care was 15.5 (7.77) days. The majority of the caregivers expressed a zero WTP (263, 61.88%) and WTA 243 (51.18%) that were mostly protest zeros [147 out of 263 (55.89%] WTP and 213 out of 243 (87.65%) WTA. The total means (SD) of WTP and WTA for one hour more/ less caring were $12.02 (28.73), and $10.30 (15.43) USD respectively. The economic burden is calculated by purchasing power parity (PPP) factor in 2021.

The total economic burden of informal care was calculated at $ 566,132.94 and $ 467,084.02 USD based on WTP and WTA approaches (Table 2).

**Table 2:** Economic burden (in $ USD) of informal care for the COVID-19 patients’ caregivers

	WTP	WTA
Total mean (SD) including zero responses ($)	12.02(28.73)	10.30(15.43)
Mean (SD) excluding zero responses ($)	15.88(18.42)	24.4(15.01)
Number of positive values (%)	182(42.82)	182(42.82)
Number of zero values (%)	243(57.18)	243(57.18)
Number of real zero values (%)	29(11.93)	30(12.34)
Number of protest zero values (%)	214(88.06)	213(87.65)
Total economic burden ($)	566,132.94	467,084.02

Based on the double hurdle regressions, in the selection equations, by increasing the patients’ age the probability of reporting a positive WTP and a positive WTA increased [OR = exp(0.001) = 1.001, OR = exp(− 0.012) = 0.988 respectively]. However, this variable was not included in the any of quantity equations of both regressions.

Caregivers’ age showed no statistically significant relationship to the probability of reporting positive WTP/WTA (p-value = 0.886, p-value = 0.060 respectively). In addition, the mean of lnWTP/lnWTA did not change statistically significantly with the caregivers' age (p-value = 0.307, p-value = 0.363).

Being employed by the caregiver during giving care to patients increased the probability of reporting positive WTP [OR = exp(0.555) = 1.74]. In the second regression, caregivers who were employed reported a higher lnWTP mean (p-value = 0.044). Employment of caregivers increased the lnWTP by 0.042 Tooman. Although, like WTP, employing caregivers increased the probability of reporting positive WTA [OR = exp(0.469) = 1.599], being employed did not change the mean of lnWTA in the second equation of the WTA regression (p-value = 0.058).

The caregiver whose patient was her/his spouse/child was more likely to report a positive WTP/WTA [OR = exp(0.244) = 1.276, OR = exp(0.682) = 1.979 respectively]. This variable was not included in the second equation of both regressions.

By increasing the number of caring days, the probability of reporting positive WTP did not change statistically significantly (p-value = 0.753.), but it was less likely that the caregivers reported a positive WTA (OR = exp(− 0.031) = 0.969). This variable was present in both quantity equations and only in the WTP regression by increasing the caring day by one day, the mean of ln WTP increased by 0.004 Tooman (p-value < 0.0001).

Caregiver sex was included in the first equation of both regressions and only statistically significantly being men compared to women decreased the probability of reporting positive WTA [OR = exp(0.042) = 1.043].

Perceived difficulty in doing outdoor activities compared to personal activities decreased the mean of lnWTP by − 0.048 (p-value = 0.002). Regarding WTA, difficulty in doing indoor activities decreased the mean of lnWTA by − 0.027 (p-value = 0.043). Other variables did not show any statistically significant relationship with the dependent variables in both regressions (Tables 3, 4).

**Table 3:** Two parts regression of the caregivers’ willingness to pay

	Part 1: probability of WTP > 0 (N = 425)		Part 2: amount lnWTP given lnWTP > 0 (n = 182)	
	Estimate (SE)	p-value	Estimate (SE)	p-value
Intercept	− 1.054(0.309)	0.001^**^	0.784(0.043)	0.000^***^
Age of patient	0.007(0.003)	0.012^*^	–	–
Age of care giver	0.001(0.006)	0.886	0.001(0.001)	0.307
Gender of care giver (ref^a^: female)				
Male	0.042(0.099)	0.669	–	–
Care giver history of getting COVID-19 (ref: yes)				
No	0.089(0.138)	0.519	0.008(0.019)	0.666
Receiving formal care by patient (ref: yes)				
No	0.006(0.148)	0.970	0.002(0.020)	0.904
Job of care giver (ref: unemployed)				
Employed	0.555(0.135)	0.000^***^	0.042(0.021)	0.044^*^
Relationship of patient with care giver (ref: spouse/child)				
Other	0.244(0.096)	0.011^*^	–	–
Number of care days	0.002(0.008)^**^	0.753	0.004(0.001)	0.000^***^
Education of care giver (ref: lower than undergraduate)				
≥ Undergraduate	–	–	0.005(0.016)	0.745
Difficulty of activities (ref: personal activities)				
Indoor activities	–	–	− 0.022(0.012)	0.076^†^
Outdoor activities	–	–	− 0.048(0.015)	0.002^**^
Taking patients to the formal care centers	–	–	− 0.007(0.022)	0.764

^†^p < 0.1, *p < 0.05, **p < 0.01, ***p < 0.001, a: reference

**Table 4:** Two parts regression of the caregivers’ willingness to accept

	Part 1: probability of WTA > 0 (N = 425)		Part 2: amount lnWTA given lnWTA > 0 (n = 182)	
	Estimate(SE)	p-value	Estimate(SE)	p-value
Intercept	− 0.389(0.379)	0.305	1.003(0.022)	0.000^***^
Age of patient	0.018(0.005)	0.000^***^	–	-
Age of care giver	− 0.012(0.006)	0.060^†^	0.000(0.000)	0.363
Gender of care giver (ref: female)				
Male	− 0.569(0.166)	0.001^***^	–	-
Care giver history of getting COVID-19 (ref: yes)				
No	0.012(0.147)	0.934	0.002(0.009)	0.851
Receiving formal care by patient(ref: yes)				
No	− 0.029(0.145)	0.843	0.008(0.008)	0.360
Job of care giver (ref: unemployed)				
Employed	0.469(0.161)	0.004^**^	− 0.016(0.008)	0.058^†^
Relationship of patient with care giver (ref: spouse/child)				
Other	0.682(0.185)	0.000^***^	–	–
Number of care days	− 0.031(0.009)	0.001^**^	0.001(0.001)	0.141
Dependency of patient to care giver(ref: completely)				
< completely	− 0.141(0.144)	0.326	− 0.008(0.008)	0.324
Education of care giver (ref: lower than undergraduate)				
≥ undergraduate	–	–	0.015(0.009)	0.104
Difficulty of activities (ref: personal activities)		–		
Indoor activities	–	–	− 0.027(0.013)	0.043^*^
Outdoor activities	–	–	− 0.016(0.014)	0.246
Taking patients to the formal care centers	–	–	− 0.029(0.015)	0.061^†^

^†^p < 0.1, *p < 0.05, **p < 0.01, ***p < 0.001

## Discussion

Informal care could play a significant role in mitigating the COVID-19 burden on health systems; however, this care put pressure on caregivers. Identifying driving factors in the tendency to provide such critical care could empower health systems in the pandemic management. To this end, it is necessary to elicit the value of this care for their providers, extract the driving factors behind their tendency for providing informal care, and in the next step plan for the support of caregivers. The present study aimed to identify variables related to WTP/WTA for informal care and quantify the economic value of this care in Sanadaj city, Iran.

In this study, the probability of reporting a positive WTP/WTA increased by increasing the patients’ age. Other studies revealed diverse findings [16–19]. In one study, patients' age showed no statistically significant relationship with the probability of reporting positive WTP and with the amount of WTP [18]. By increasing the patient's age, probably caregivers would face more difficulty in caring, which in turn they could reveal a more willingness to delegate care to others. In terms of WTA, the caregivers showed more tendency to accept money in exchange for taking care of older patients as their perceived burden of caring increased, and so does the caregiver's willingness to accept [20, 21]. Other studies have reported no significant relationship between caregivers’/patients' age and caregivers’ WTA [17, 22]. By increasing the patient's age and probably the increased severity of the disease, there could be a higher level of physical and mental burden which could be related to the direct relationship between the perceived burden of patient care and the caregiver’s WTA [11, 23].

In line with our findings, there was no relationship between caregivers’ age and WTP [24, 25]/WTA [17, 22]. Although a significant negative association has been observed in other studies between caregivers’ age and their WTP [16, 18]/WTA [26].

Holding an academic background could be related to the increased probability of reporting a positive WTP and a higher positive amount of WTP [25], in this study, no statistically significant relationship was found between the caregivers' education level and their lnWTA/lnWTP that this is in consistency with another research addressing WTA [20].

In addition, the patient’s dependency on the caregiver showed no statistically significant relationship to the caregivers' WTA (in both equations). Other studies showed that by increasing the patient's dependency on the caregiver, caregivers’ WTP increased too [27], however in this study, this variable was not included in the WTP regressions.

Consistently previous research found no statistically significant relationship between caregivers’ gender and their WTP [17, 22, 24]; while similar to some other studies [17, 26] and quite opposite to other research [20, 22], men showed less probability/amount of WTA. The differences in the gender groups’ attitudes toward their ability to care for patients over a longer period could explain men’s lower tendency in providing informal care [26]. In other words, by decreasing the caregiver’s confidence in his/her ability in caring, there could be a decreased WTA too [20]. In addition, men’s work situation and employment in higher-paying jobs, compared to women [28], means that they probably must waive more income to provide informal care which could explain their lower WTA.

Employed caregivers were more likely to report a positive WTP and reported a higher lnWTP amount. Jetquitz et al. obtained inconsistent results; in their study, unemployed caregivers reported a higher WTP. However, regarding the probability equation, they did not find any statistically significant relationship [25]. Higher payment capacity in employed individuals compared to non-employed ones can explain this finding. Being in the higher-income and socio-economic classes is related to reporting an increased WTP [29]. Restrictions on work absenteeism, risk of losing the job, and higher opportunity costs in employed individuals [26, 28, 30] are other possible reasons. The positive relationship between caring for patients and having problems in the workplace by caregivers has been confirmed [31, 32]. Employed caregivers were more likely to accept money; however, the magnitude of their acceptance was not statistically different from the unemployed caregivers. Montazakis et al. also confirmed a higher opportunity cost and an increased WTA in employed caregivers [26]. However, another study found more WTA in housewives and less WTA in caregivers working in flexible jobs [20].

The majority of the caregivers believed that the two most difficult tasks were the indoor and the outdoor activities in terms of the consumed time amount followed by the personal activities and finally companying the patients to formal care centers. Similarly, Liu et al.’s found that indoor activities were the most difficult activity [16]. Doing the patient's outdoor activities was related to a lower lnWTP compared to the reference level (doing the patient's personal activities). De Meijer et al. showed that the activity type had a statistically significant relationship with the caregivers’ WTP [17]. Liu et al. revealed that WTP was not related to the type of activities; however, their analysis was not adjusted for other variables and the relationship between the type of difficult activity and WTP was not assessed [16]. Doing indoor activities also decreased the amount of lnWTA compared to personal activities. Liu et al. found that chores were the most difficult activities for caregivers and increased the caregivers' WTA [16]. Another study did not reveal any statistically significant relationship between the number of activities and the caregivers’ WTA, but when they considered the type of activities, the relationship was statistically significant [17]. Previously we assumed that the caregivers would need more compensation for the most difficult activities, but surprisingly doing such activities decreased the mean of lnWTP/lnWTA. Considering process utility theory, individuals gain utility from the process of activities [33]. Although the most difficult activities were indoor and outdoor activities respectively, being in close touch with the COVID-19 patients through personal activities probably played a more important role in the caregivers' valuation. In other words, caregivers may prefer to spend more time on safer activities (such as indoor and outdoor activities) than doing risky tasks such as patients’ personal activities. So it is accepted that they have reported a lower WTP /WTA for these safer activities.

The results showed no statistically significant relationships between the history of caregivers' infection to COVID-19 and their willingness to report a positive WTP/WTA and their lnWTP/lnWTA amount.

A higher probability of reporting positive WTP /WTA was obtained when the patient was not the caregiver’s spouse/child. Other studies revealed that caregivers who provided care to their spouse/child had a lower WTP [20, 24]. Gervès-Pinquié, et al. showed that being the spouse/parents of patients could increase the amount of WTP, but the probability of reporting a positive WTP did not change. Having more committed to taking care of close relations could explain this finding [18]. De Meijer et al. also revealed an increased WTP in the caregivers who took care of their spouse/child [17]. Concerning WTA, van denBerg et al. did not find any statistically significant relationship which is not in line with this study's findings [20]. Mentzakis et al. revealed that being a spouse/child of caregivers was related to a higher WTA [26]. Social and cultural differences could explain discrepancies between the present study and other findings.

An increase in the number of caring days which could bring more burden and stress for the caregivers [32, 33] had a positive relationship with the mean of lnWTP. However, the probability of positive WTP did not change significantly. De Meijer et al. reported similar findings [17]. Gervès-Pinquié et al. did not find any significant relationship [18]. The probability of reporting a positive WTA was negatively related to the number of caring days, however, the amount of lnWTA showed no significant relationship with the number of caring days. Other studies showed diverse results [17, 20, 22]. It seems a prolonged caring duration [16], caused more disturbance in caregivers’ personal life, which in turn decreased their WTA.

Although it was expected that formal care and informal care substitute each other, to some extent [34], there was no statistically significant relationship between patients’ history of formal care utilization and the caregivers’ WTP/WTA in none of the equations. Gervès-Pinquié, et al. showed that caregivers whose patients have such experiences were more likely to report a positive WTP [18]. The extent of the substitution between formal and informal care is related to having strong family bonds and social norms [32], it seems in the present study the effects of these factors were almost the same in all caregivers.

Due to ethical and humanitarian reasons, most participants reported zero responses for WTP/WTA. By the way, comparatively discussing, the average WTP was more than the average WTA which is similar to another study [35]. This finding could root in the perceived advantage of caring for loved ones [35] and the caregivers' preferences for getting COVID-19 care from educated caregivers [7]. In addition, it seems in the present study, culturally, it was more acceptable to pay for informal care compared to accepting money. Other studies reported a higher mean for WTA [16, 17]. The total economic burden of informal care based on the WTP approach was higher than the WTA. Another study on Alzheimer showed that the burden of the disease is different for caregivers depending on the used valuation method [36].

Although the present study was the first study that calculated the economic burden of informal care on the COVID-19 patients’ caregivers and determined the influential factors in their valuation, there are still some limitations that are worth mentioning. Considering the difficulty in interviewing the caregivers, an open end format was used; it seems using other formats could provide different results. Assessing caregivers’ mental health could provide precious information about the mental pressures that resulted from caring for COVID-19 patients which were not assessed in this study.

## Conclusion

Caring for COVID-19 patients could have positive and negative aspects. Humanitarian feelings could bring utility for caregivers; while physical and mental pressures resulting from COVID-19 could negatively affect their well-being. Designing and implementing supportive plans for caregivers need to identify the motivators of their involvement in the caring process. This study revealed that the utilization of the priceless capacity of informal care is mostly related to the caregivers’ employment status, their gender, their perceived difficulty in doing various activities, and the caring duration. Increasing caregivers’ self-efficacy and facilitating their involvement in the caring process could be achieved through flexible work status, educational programs, and other interventions to reduce their burnout.

## Data Availability

The datasets used and/or analyzed during the current study are available from the corresponding author upon reasonable request.

## Abbreviations

COVID-19: Coronavirus disease of 2019
exp: Exponential
ICU: Intensive Care Unit
lnWTA: Natural logarithm of Willingness To Accept
lnWTP: Natural logarithm of Willingness To Pay
OR: Odds Ratio
ref: Reference
SD: Standard Deviation
WHO: World Health Organization
WTA: Willingness to accept
WTP: Willingness to pay
